# HIV prevalence among high school learners - opportunities for schools-based HIV testing programmes and sexual reproductive health services

**DOI:** 10.1186/1471-2458-12-231

**Published:** 2012-03-22

**Authors:** Ayesha BM Kharsany, Mukelisiwe Mlotshwa, Janet A Frohlich, Nonhlanhla Yende Zuma, Natasha Samsunder, Salim S Abdool Karim, Quarraisha Abdool Karim

**Affiliations:** 1 Centre for the AIDS Programme of Research in South Africa (CAPRISA), 2nd Floor, Doris Duke Medical Research Institute, Nelson R Mandela School of Medicine, University of KwaZulu-Natal, Private Bag 7, Congella, Durban 4013, South Africa; 2 Department of Epidemiology, Mailman School of Public Health, Columbia University, New York, USA

**Keywords:** Young girls, HIV prevalence, surveillance

## Abstract

**Background:**

Young girls in sub Saharan Africa are reported to have higher rates of human immunodeficiency virus (HIV) infection compared to boys in the same age group. Knowledge of HIV status amongst high schools learners provides an important gateway to prevention and treatment services. This study aimed at determining the HIV prevalence and explored the feasibility of HIV testing among high school learners.

**Methods:**

Between September 2010 and February 2011, a linked, anonymous cross-sectional survey was conducted in two public sector high schools in the rural KwaZulu-Natal midlands. Following written informed consent, dried blood spot samples (DBS) were collected and tested for HIV. The overall and age-specific HIV prevalence were compared with select demographic variables.

**Results:**

The HIV prevalence in learners aged 12 to 25 in school A was 4.7% (95% CI 2.8-6.5) compared to 2.5% (95% CI 1.6-3.5) in school B, (p = 0.04). Whilst the HIV prevalence was similar for boys at 1.3% (95% CI 0-2.8) in school A and 1.7% (95% CI 0.5-2.8) in school B, the prevalence in girls was consistently higher and was 7.7% (95% CI 4.5-10.9) in school A and 3.2% (95% CI 1.8-4.6) in school B. The age-specific HIV prevalence in girls increased 1.5 to 2 fold for each two year age category, while for boys the prevalence was stable across all age groups.

**Conclusions:**

The high HIV prevalence in female learners underscores the importance of sexual reproductive health and schools-based HIV testing programs as an important gateway to prevention and treatment services.

## Background

In generalized HIV epidemic settings, anonymous testing of pregnant women attending public sector antenatal clinic (ANC) remain the mainstay for monitoring epidemic trends and are important for measuring the magnitude of the epidemic in the general population [1]. The Joint United Nations Programme on HIV/AIDS (UNAIDS) recommends the importance of understanding the HIV epidemic locally and customizing responses accordingly [2]. As part of the ongoing research conducted by the Centre for the AIDS Programme of Research in South Africa (CAPRISA) in the sub-district of Vulindlela we have monitored the trends in HIV prevalence in this rural community through the annual ANC HIV seroprevalence surveys in the seven primary health care clinics [3]. These surveys have coincided with the national department of health ANC surveys [4] and have revealed a disturbing increase in HIV prevalence among young women below 20 years of age. The overall HIV prevalence in this age group increased from 16.6% in 2006 to 20.8% in 2008 and the HIV prevalence in this age group serves as a reliable proxy marker for incident HIV infections [1,3,4].

Whilst South Africa is one of few countries that has a supportive legal framework to enable young children to access sexual reproductive health services autonomously from age 12 [5], there are many ethical and programmatic challenges to HIV testing of young children for care and support or for surveillance. In recognition of the fact that children are sexually active at a very young age the risk of HIV acquisition increases through complex individual behaviours and through sexual networks within a broader context of political, economic and social powers. To understand the evolving HIV epidemic in this rural setting and to establish whether the prevalence in young pregnant girls is similar to that in other non-pregnant young people in this age group we undertook an anonymous survey in high school learners in Vulindlela, KwaZulu-Natal

## Methods

### Setting

The study was conducted in the rural district of Vulindlela, located 150 km north-west of Durban between September 2010 and February 2011. The community has limited infrastructure and few employment opportunities, resulting in high levels of poverty. Health services are provided by seven public sector primary health care clinics and the closest referral hospitals are approximately 30 kilometres away.

There are a total of 75 schools in this sub-district with a learner population of 42,152. Of these, 51 (68%) are primary schools with a learner population of 25,606 from grades R to 7. The remaining 24 (32.0%) are secondary schools with a learner population of 16,546 in grades 8 to 12. In preparation for the survey several consultative meetings were held with the Department of Education at the provincial, district and school level. At the school level, discussions were held with principals and educators, schools governing bodies, parents and learners prior to implementation.

### Study design

This cross-sectional, anonymous, linked survey was undertaken in two, randomly selected high schools in Vulindlela. Each school was visited over a three week period by a team of trained nurses and counsellors. Information on the purpose of the study, the implications of participation, the informed consent process, sample collection procedures, use of the results and confidentiality of data were provided to learners in small groups of 10 to 15. Following individual informed consent, learners willing to participate in the survey provided specimens for HIV testing. Blood was collected through finger-prick onto blotting paper [Dried blot specimen (DBS)] [6]. A limited set of demographic information, identified only by a unique study code, was linked to the DBS sample. No personal identifying information was used to ensure confidentiality. The HIV test results were matched to demographic data by their unique codes.

All learners were provided with information on how to access HIV testing and care services and HIV risk reduction counselling including access to medical male circumcision for male learners. Learners were encouraged to inform family members on the availability of HIV counselling and testing services and where to access these in the district.

### Study approvals

The Biomedical Research Ethics Committee of the University of KwaZulu-Natal (E179/04) and the KwaZulu-Natal Departments of Health and Education reviewed and approved the study.

### HIV serological testing

HIV testing was undertaken using HIV ELISA testing performed on DBS samples for detecting antibodies to HIV using the Vironostika Uni-Form 11 plus O Assay, Biomerieux, with results being recorded as negative or positive.

### Data management and statistical analysis

The survey was undertaken in the schools among all registered learners to minimize selection and participation bias. Data were collected on standardized case report forms (CRFs) and faxed to a dedicated study database using DataFax (Clinical DataFax Systems Inc., Hamilton, Canada). HIV and demographic data analysis were undertaken using the SPSS software version 18.0. Individual sample weights were weighted to generate a final weighted sample that closely matched the 2010 mid-year population estimates for the province of KwaZulu-Natal provided by Statistics South Africa [7]. The Fisher's exact test was used to test for differences in HIV prevalence. The 95% confidence interval (CI) for the crude and weighted HIV prevalence assumed a Poisson distribution.

## Results

A total of 492/696 learners in school A and 1074/1150 learners in school B participated in the study giving a response rate of 71.0% and 93.4% respectively. The mean age of learners at both schools was similar and ranging from 12 to 25 years. At both schools more girls were attending school than boys and the number of learners in each grade varied per school. The majority of learners were in grades 8, 9 and 10 with a substantial decline in number of girls and boys in grades 11 and 12 (Table 1).

**Table 1:** Demographic characteristics and HIV prevalence among learners in rural KwaZulu-Natal, South Africa

Variable	School A (N = 492)					School B (N = 1074)				
	**Boys**		**Girls**			**Boys**		**Girls**		

***Demographic characteristics***										

**Age**										

Age (Mean; ± SD; range)	16.4; ± 2.2;13-24		16.6; ± 2.4;12-25			15.7; ± 2.2;12-23		15.1; ± 2.0;12-22		

**Age groups**	**n**	**%**	**n**	**%**		**n**	**%**	**n**	**%**	

12-14 years	40	17.4	62	23.8		151	31.7	265	44.6	

15-16 years	89	38.7	76	29.2		172	36.1	186	31.3	

17-18 years	62	27.0	70	26.9		100	21.0	102	17.2	

19-25 years	39	17.0	50	19.2		54	11.3	41	6.9	

**Overall**	**230**	**(46.9)**	258*	**(53.1)**		477*	**(44.5)**	594*	**(55.5)**	

**Grade distribution**										

Grade 8	62	27.0	74	28.5		105	22.0	141	23.7	

Grade 9	66	28.7	46	17.7		125	26.2	139	23.3	

Grade 10	57	24.8	48	18.5		119	24.9	156	26.2	

Grade 11	28	12.2	37	14.2		88	18.4	109	18.3	

Grade 12	17	7.4	55	21.2		41	8.6	51	8.6	

**Overall**	**230**	**(46.7)**	260*	**(52.8)**		**478**	**(44.5)**	**596**	**(55.5)**	

***HIV prevalence***										

	**n**	**%95%CI**	**n**	**%95%CI**		**n**	**%95%CI**	**n**	**%95%CI**	

**Age specific prevalence**										

12-14 years	2	5.0(0.4-18.2)	1	1.6(0.1-9.8)		0	0(0.1-3.1)	5	1.9(0.7-4.6)	

15-16 years	1	1.1(0.1-7.0)	2	2.6(0.5-10.1)		2	1.2(0.2-4.6)	5	2.7(1.0-6.5)	

17-18 years	0	0 (0.2-7.3)	5	7.1(2.7-16.6)		4	4.0(1.3-10.5)	4	3.9(1.3-10.5)	

19-25 years	0	0(0.2-11.2)	12	24.0(13.5-36.5)		2	3.7(0.6-13.8)	5	12.2(4.6-27.0)	

**Grade specific prevalence**										

Grade 8	3	4.8(1.3-14.4)	2	2.7(0.5-10.3)		1	1.0(0.1-6.0)	2	1.4(0.3-5.6)	

Grade 9	0	0 (0.1-6.9)	3	6.5(1.7-18.9)		2	1.6(0.3-6.2)	5	3.6(1.3-6.6)	

Grade 10	0	0 (0.2-7.9)	0	0 (-)		1	0.8(0.1-5.3)	6	3.8(1.6-8.6)	

Grade 11	0	0 (0.3-15.0)	7	18.9(8.6-35.7)		3	3.4(0.9-10.3)	6	5.5(2.3-12.1)	

Grade 12	0	0 (0.5-22.9)	8	14.6(6.9-27.2)		1	2.4(0.1-14.4)	0	0(0.2-8.7)	

										

**Crude HIV prevalence**	**1.3% (95% CI 0-2.8)**		**7.7% (95% CI 4.5-10.9)**		P < 0.001	**1.7% (95% CI 0.5-2.8)**		**3.2% (95% CI 1.8-4.6)**		P = 0.12

Weighted HIV prevalence**	**1.4% (95% CI 0-2.8)**		**12.7% (95% CI 7.5-19.3)**			**2.5% (95% CI 0.1-5.0)**		**7.0% (95% CI 2.4-12.2)**		

**Crude HIV prevalence**		**4.7% (95% CI 2.8-6.5)**					**2.5% (95% CI 1.6-3.5)**			

Weighted HIV prevalence**		**7.6% (95%CI 4.3-10.9)**					**4.5% (95%CI 2.1- 6.9)**			

* Missing values were excluded from percentage calculation

** weighted to the 12-24 year old 2010 mid-year population estimates for the province of KwaZulu-Natal provided by Statistics South Africa [7].

**Figure 1:**
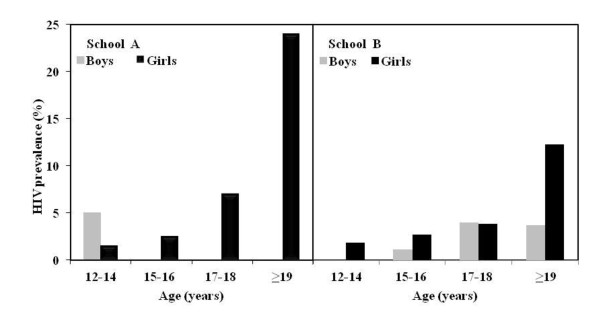
**Age-specific HIV prevalence for boys and girls in School A and in School B in rural KwaZulu-Natal, South Africa**.

The overall HIV prevalence in learners in school A was 4.7% (95% CI 2.8-6.5), which was significantly higher than the prevalence of 2.5% (95% CI 1.6-3.5) in school B, (p = 0.04). The overall HIV prevalence in girls was consistently higher at 7.7% (95% CI 4.5-10.9) in school A and 3.2% (95% CI 1.8-4.6) in school B compared to the prevalence in boys at 1.3% (95% CI 0-2.8) in school A and 1.7% (95% CI 0.5-2.8) in school B. While the prevalence in girls at the two schools differed significantly (7.7% versus 3.2%; p = 0.0006), the age-specific HIV prevalence increased 1.5 to 2 fold for each two year age category. For boys, however, the HIV prevalence remained stable across all age categories (Figure 1).

## Discussion

The HIV prevalence among high-school learners in this rural district is concerning and underscores the importance of targeted HIV risk reduction and sexual reproductive health service efforts in high schools. It is also important to better understand why these young girls are having sex, with whom and when they are having sex to better inform efforts to reduce their HIV risk. Early sexual debut is associated with higher teenage pregnancy rates, sexually transmitted infections and poor school completion rates leading to poor health and economic outcomes in young women [8-12].

South Africa is one of few countries that has a supportive legal framework to enable young children to access sexual reproductive health services autonomously from age 12 [5]. The Children's Act of 2005 (Act No. 38 of 2005) which came into effect in 2007 explicitly allows children 12 years and older access to contraceptives, information on sexuality and reproduction, and the right of consent to HIV/AIDS testing and treatment. This is in recognition of the fact that children are sexually active at a very young age and that there is a high burden of HIV infection in young people [13], despite the legal age of consent for sex being 16 years.

The high HIV prevalence among girls identified through this study suggests that these infections are likely to remain undiagnosed, sustaining the networks for HIV transmission. In spite of high levels of awareness on the benefits of HIV testing, fear of HIV-related stigma from parents, caregivers and teachers could be barriers preventing learners from being tested [14-16]. In high HIV prevalence settings, there is a need for more comprehensive information and support on HIV-related issues, including the importance of prevention.

The large gender disparity in the sample surveyed showed that the HIV prevalence was substantially lower in young boys compared to young girls. Young boys were significantly less likely to be HIV infected than their female counterparts; however, the prevalence in older boys was high. The patterns of infection observed in young girls are similar to trends observed throughout southern Africa, although these are restricted to age groups of 15 years and older [6,9,17]. Several large studies focusing on knowledge of HIV and behavioural risk factors for sexually transmitted infections and HIV have been conducted in the absence in biological measurements. These studies have demonstrated increasing risk taking behaviour predisposing to infections [18,19]. As early adolescent's marks a critical time of physical, developmental and social changes, interventions must focus on the needs of young people from as early as 10 years of age. These interventions need to be effective in shaping healthy attitudes and behaviours while most learners are still at school and less likely to have begun sexual activity.

As young people in this country are becoming sexually active at younger ages, it is important that they are taught that regular HIV testing should be an important part of their routine healthcare. While many social barriers are expected, by engaging each other openly, honestly and directly, young people would have an opportunity to impact on sexual health choices. The HIV and STI risk reduction programmes at schools are crucial in moulding and developing young people's identities and characters to enhance self esteem and thereby reduce or delay early sexual debut. Furthermore these choices would enable young people to reduce their anxiety, fear and stigma associated with HIV testing especially as they move into adulthood and the risk of infection increases, even before they are sexually active.

These results demonstrate the growing need and the opportunities for HIV testing and sexual reproductive health services within the school setting. South Africa has a high teenage pregnancy rate and in 2003 an estimated 12% of teenage girls between the ages of 15 and 19 years had ever been pregnant or were pregnant at the time of the survey [20]. The most recent reports show that the learner pregnancy rate in KwaZulu-Natal was 62.2 per 1000 [21]. Despite a decade of increased spending on sex education and HIV/AIDS awareness campaigns, there has been little impact on pregnancy rates and HIV incidence.

Learners as young as 12 years were included in this study because early age of sexual debut is considered a crucial factor affecting the vulnerability of young people to HIV infection. Several studies have found that sexual debut before the age of 15 years to be approximately 10% for both boys and girls [22-25]. This figure increases substantially following experimentation with alcohol, substance abuse, pressure from mixing with older peer groups, coercion or sexual abuse. Young people initiating sex at an early age has major implications for HIV and STI infection and associated with higher HIV exposure due to it being linked to more frequent sexual intercourse, more lifetime sexually transmitted infections, less consistent contraceptive use, and more sexual partners [26].

The National Minister of Health of South Africa recently announced the prioritization of schools for the HIV counselling and testing (HCT) campaigns [27]. The close partnerships between the government departments and the community that will be necessary to successfully implement this campaign will go a long way towards addressing the challenges of obtaining consent, maintaining confidentiality, and providing the psychological and emotional support for young people to deal with an HIV-positive diagnosis. The magnitude of the epidemic demands more HIV risk reduction efforts in schools with evidence based programs.

There are several limitations to our study. The small sample size limits representativeness and generalizability to the larger school population in the district. Furthermore, the schools learner populations may differ by schools and the geographic variability in the prevalence of HIV infection is likely to be different across the district. Nevertheless this study is the first to report on the prevalence of HIV in high school learners in Vulindlela. These learners represent a population of young people with emerging HIV epidemic. Therefore, surveillance is a necessary tool to understand the transmission dynamics and the evolving epidemic in this vulnerable group. Another limitation was the absence of behavioural data collection which limited our understanding of risk behaviours and on the modes of HIV transmission. Despite these limitations, our findings indicate that additional larger studies are required to determine the full meaning of the alarming HIV prevalence in the sample of young women.

## Conclusions

Knowledge of HIV status provides an important gateway to prevention and care efforts and the HCT campaign in schools could provide a narrow window of opportunity to reduce HIV risk in young girls and stem the tide of HIV for this and future generations.

## Competing interests

The authors declare that they have no competing interests.

## Authors' contributions

ABMK took lead in preparing the manuscript. She participated in the proposal development, study design, project implementation, supervision, overall project management, analysis, and interpretation of results. MM participated in supervision and project implementation. JAF participated in the proposal development, study design, project implementation and manuscript preparation. NS co-ordinated the laboratory testing and NYZ conducted the statistical analysis. QAK and SSAK participated in the interpretation of results and writing of the manuscript. All authors read and approved the final manuscript.

## Pre-publication history

The pre-publication history for this paper can be accessed here:

http://www.biomedcentral.com/1471-2458/12/231/prepub
